# A subgroup I bZIP transcription factor PpbZIP18 plays an important role in sucrose accumulation in peach

**DOI:** 10.1186/s43897-025-00156-0

**Published:** 2025-07-03

**Authors:** Xian Zhang, Wen Xiao, Yudi Liu, Yunpeng Cao, Ruo-Xi Zhang, Yuepeng Han

**Affiliations:** 1 State Key Laboratory of Plant Diversity and Specialty Crops, Wuhan Botanical Garden, Chinese Academy of Sciences, Wuhan, 430074 China; 2 Hubei Hongshan Laboratory, Wuhan, 430070 China; 3 University of Chinese Academy of Sciences, Beijing, 100049 China; 4 Department of Plant Sciences, State Key Laboratory of Hybrid Rice, College of Life Sciences, Wuhan University, Wuhan, 430072 China

Peach (*Prunus persica*) is a widely cultivated fruit tree species in temperate regions. Sugar content is a key indicator in evaluating the flavor quality of peaches, and fruits with high sugar levels exhibit a more intense flavor, thus highly preferred by consumers. Sugar accumulation is a complex biological process primarily involving sugar synthesis and transport, yet its related mechanism remains largely unclear in peach.

The *bZIP* transcription factors (TFs) in subgroups A and S are known to regulate fruit sugar accumulation (Ma et al. 2017; Li et al. 2021; Wang et al*.*, 2023). Analysis of *bZIP* TFs in the peach genome revealed one member (Prupe.6G041400) designated PpbZIP18, which is located at 3.06 Mb on chromosome 6 within a previously reported quantitative trait locus (QTL) controlling soluble solid content (Hernández Mora et al. 2017). Analysis of our previously reported peach fruit transcriptomes (Zheng et al. 2021) showed that the expression profile of *PpbZIP18* was consistent with the accumulation patterns of sucrose (*r* = 0.89, *P* < 0.01) and total soluble sugars (*r* = 0.92, *P* < 0.01) (Figure S1A). Transient overexpression or silencing of *PpbZIP18* caused significant changes in the accumulation of sucrose and total soluble sugars, but not glucose and fructose in peach fruits (Fig. 1A, B; Figure S1B, C). Ectopic overexpression of *PpbZIP18* in tomato resulted in a significant increase in total sugar content in fruits after breaker (Figure S2). Notably, PpbZIP18 belongs to the bZIP TF subgroup I (Figure S1D), which has not been reported to regulate sugar accumulation. These findings prompted us to investigate the mechanisms by which PpbZIP18 regulates sugar accumulation.

**Fig. 1:**
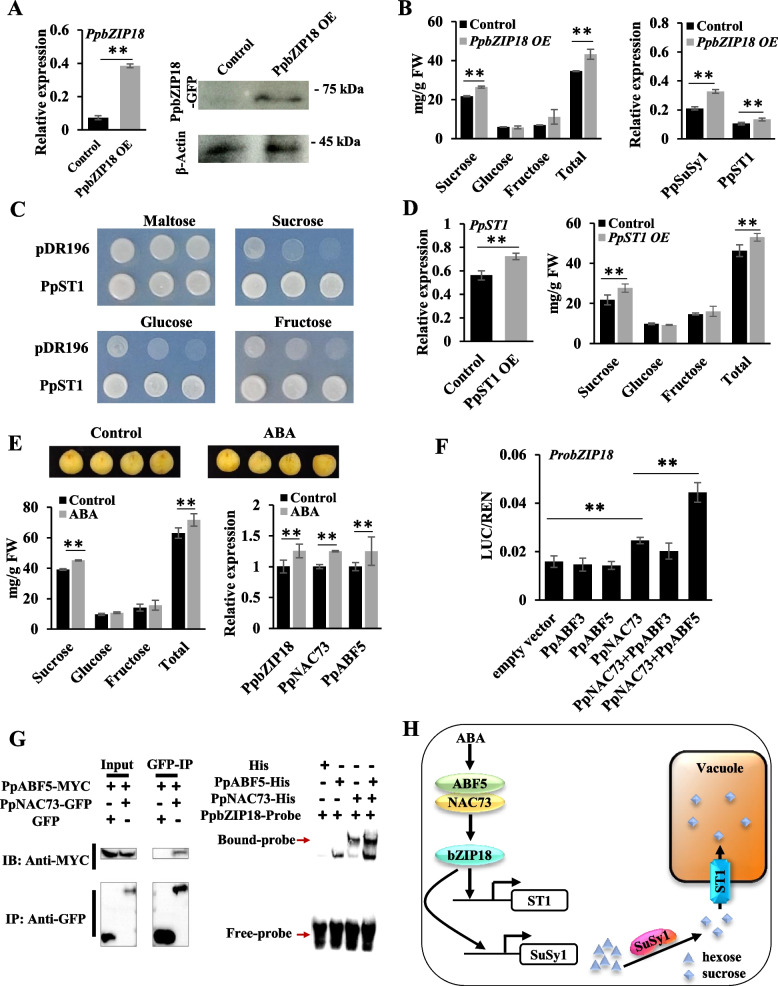
The regulatory role of *PpbZIP18* in sugar accumulation in peach fruit. **A** The transcript and protein levels of *PpbZIP18* in peach fruits transiently overexpressing *PpbZIP18*. **B** The content of sugar components and the expression of *PpSuSy1* and *PpST1* in peach fruits tranisently overexpressing *PpbZIP18*. **C** Functional analysis of PpST1 in the hexose transport- and SUC2-deficient yeast strain CSY4000 that was grown in SD/-Ura medium supplemented with various sugars. **D** The expression of *PpST1* and the content of sugar components in peach fruits transiently overexpressing *PpST1*. **E** The content of sugar components and the expression of sugar regulatory genes in peach fruits at 3 days after ABA treatment. ddH_2_O was used as control. **F** Synergistic activation effect of PpABF3/5 and PpNAC73 on the *PpbZIP18* promoter. **G** Co-IP and EMSA assays showing synergistic activation effect of PpABF5 and PpNAC73 on the *PpbZIP18* promoter. **H** A proposed model for the regulatory role of *PpbZIP18* in sucrose accumulation in peach fruit. Error bars represent the standard error (*n* = 3). Asterisks denote significant differences based on Student's *t*-test. *P < 0.05, **P < 0.01

PpbZIP18 was localized at both the nuclear and plasma membranes (Figure S1E). Analysis of fruit transcriptomes revealed two genes, *PpSuSy1* and *PpST1*, which had expression profiles similar to that of *PpbZIP18* throughout fruit development (Figure S3). PpSuSy1 has been proven to catalyze sucrose synthesis (Mollah et al. 2022), while PpST1 (Prupe.3G066300) encodes a putative plastidic glucose transporter/suppressor of G protein beta1 (pGlcT/SGB1) (Figure S4A). Subcellular localization assay showed that PpST1 was located at the vacuolar membrane (Figure S4B). Yeast functional assays using the hexose transport- and *SUC2*-deficient yeast strain CSY4000 indicated that PpST1 had the ability to transport sucrose and hexose (Fig. 1C). Moreover, transient overexpression or silencing of *PpST1* in peach fruits caused a significant change in the accumulation of sucrose, but not hexose (Fig. 1D; Figure S4C).

Yeast one-hybrid (Y1H) and dual-luciferase reporter assays showed that PpbZIP18 directly bound to the promoters of *PpSuSy1* and *PpST1* to activate their transcription (Figure S5). Electrophoretic mobility shift assay (EMSA) further revealed that PpbZIP18 could bind to the AGCTGT/G motif in the promoters of *PpST1* and *PpSuSy1* (Figure S6). Therefore, these results suggest that PpbZIP18 regulates fruit sucrose accumulation via activating the expression of *PpSuSy1* and *PpST1* in peach. To our knowledge, we report for the first time the roles of the bZIP subgroup I and the pGlcT/SGB1 subfamily in sugar accumulation. The finding that *PpbZIP18* and *PpST1* affected the accumulation of sucrose rather than hexose could be explained by at least two facts. Firstly, sucrose is the predominant sugar in peach (Vimolmangkang et al. 2016). Secondly, *PpSuSy1* acts as a sucrose synthase gene controlling sucrose synthesis that is a rate-limiting step for sugar accumulation in peach fruits (Mollah et al. 2022).

It is well known that bZIP TFs are strongly induced by ABA. The expression of *PpbZIP18* exhibited a significant increase in ABA-treated fruits of peach at 3 days after treatment, with a notable surge in the accumulation of sucrose, but not hexose (Fig. 1E). This is consistent with the result that transient overexpression of *PpbZIP18* in peach fruits caused a significant increase in sucrose content. Most importantly, ABA content is low during the early stages of fruit development, and has a remarkable increase during the ripening stages in peach (Wang et al. 2019). Consistently, sucrose is rapidly accumulated during the ripening stages to become the predominant sugar in peach fruits (Monti et al. 2016; Mollah et al. 2022). These results suggest an association of *PpbZIP18* with ABA-induced sucrose accumulation in peach. However, unlike subgroup A bZIP genes *AREB/ABFs*, the *PpbZIP18* gene lacks ABA response elements in the promoter (Figure S7). Y1H library screening revealed an upstream regulator of *PpbZIP18* designated PpNAC73 (Prupe.4G053300), which contained ABA-responsive elements in the promoter (Figure S7). Consistently, the expression of *PpNAC73* was significantly upregulated in response to ABA treatment (Fig. 1E). Analysis of fruit transcriptomes (Zheng et al. 2021) showed that the expression of *PpNAC73* significantly correlated with that of *PpbZIP18* (*r* = 0.86, *P* < 0.01). A series of biochemical assays showed that PpNAC73 could promote the transcription of *PpbZIP18* by directly binding to the SNBE motif in the promoter (Figure S8).

Subgroup A bZIP-type AREB/ABFs are important ABA-signaling components. The peach genome contains seven AREB/ABF members termed PpABF1-7. Fruit transcriptome analysis and ABA treatment assay showed that PpABF1, PpABF3 and PpABF5 participated in ABA signal transduction (Figure S9). PpNAC73 interacted with PpABF3 and PpABF5, but not with PpABF1 (Figure S10). Dual-luciferase reporter assays showed that PpNAC73 could interact with PpABF5 rather than PpABF3 to exert a synergistic activation effect on the *PpbZIP18* promoter although PpABF3 or PpABF5 alone had no activation (Fig. 1F). This finding was confirmed using co-immunoprecipitation (Co-IP) assays and EMSA experiments (Fig. 1G). Transient overexpression of *PpNAC73* or *PpABF5* in peach fruits caused a significant increase in the accumulation of sucrose rather than hexose, but a significant decrease was observed for transient silencing of *PpNAC73* and *PpABF5* (Figure S11, S12). These results suggested that *PpNAC73* and *PpABF5* act as positive regulator of fruit sugar accumulation.

Transient overexpression or silencing of *PpNAC73* or *PpABF5* in peach fruits resulted in significant changes in the expression levels of *PpST1* and *PpSuSy1* (Figure S11, S12). Interestingly, both *PpST1* and *PpSuSy1* contained the SNBE and ABRE motifs in the promoter (Figure S13). Biochemical experiments showed that PpNAC73 and PpABF5 could directly bind to the SNBE and ABRE motifs, respectively, in the promoters of *PpST1* and *PpSuSy1* to activate their transcription (Figure S14, S15). Moreover, PpNAC73 could interact with PpABF5 to exert a synergistic activation effect on the *PpST1* or *PpSuSy1* promoter (Figure S14). This represents the first report on the interaction between NAC and ABF TFs involved in ABA-induced sugar accumulation.

In summary, the PpABF5/PpNAC73 complex is activated in response to ABA signaling, thereby inducing transcription of *PpbZIP18* (Fig. 1H). Then, PpbZIP18 binds to the promoters of sucrose synthase gene *PpSuSy1* and sugar transporter gene *PpST1* to activate their expression, leading to sucrose synthesis and subsequent transport into the vacuole. Our results not only clarify the relationship between ABA signaling and sugar accumulation, but also lay a foundation for further exploring the intricate relationship between hormones and sugar metabolism.

## Supplementary Information

Supplementary Material 1.

Supplementary Material 2.

Supplementary Material 3.

## Data Availability

All data are available in the manuscript.
