# Supplementary material for: A subgroup I bZIP transcription factor PpbZIP18 plays an important role in sucrose accumulation in peach
Source: Mol Hortic. 2025 Jul 3;5:36. doi: 10.1186/s43897-025-00156-0 (PMC12224830; doi:10.1186/s43897-025-00156-0)
Supplement: Supplementary file 1 — Supplementary Material 1. [file 43897_2025_156_MOESM1_ESM.pdf]

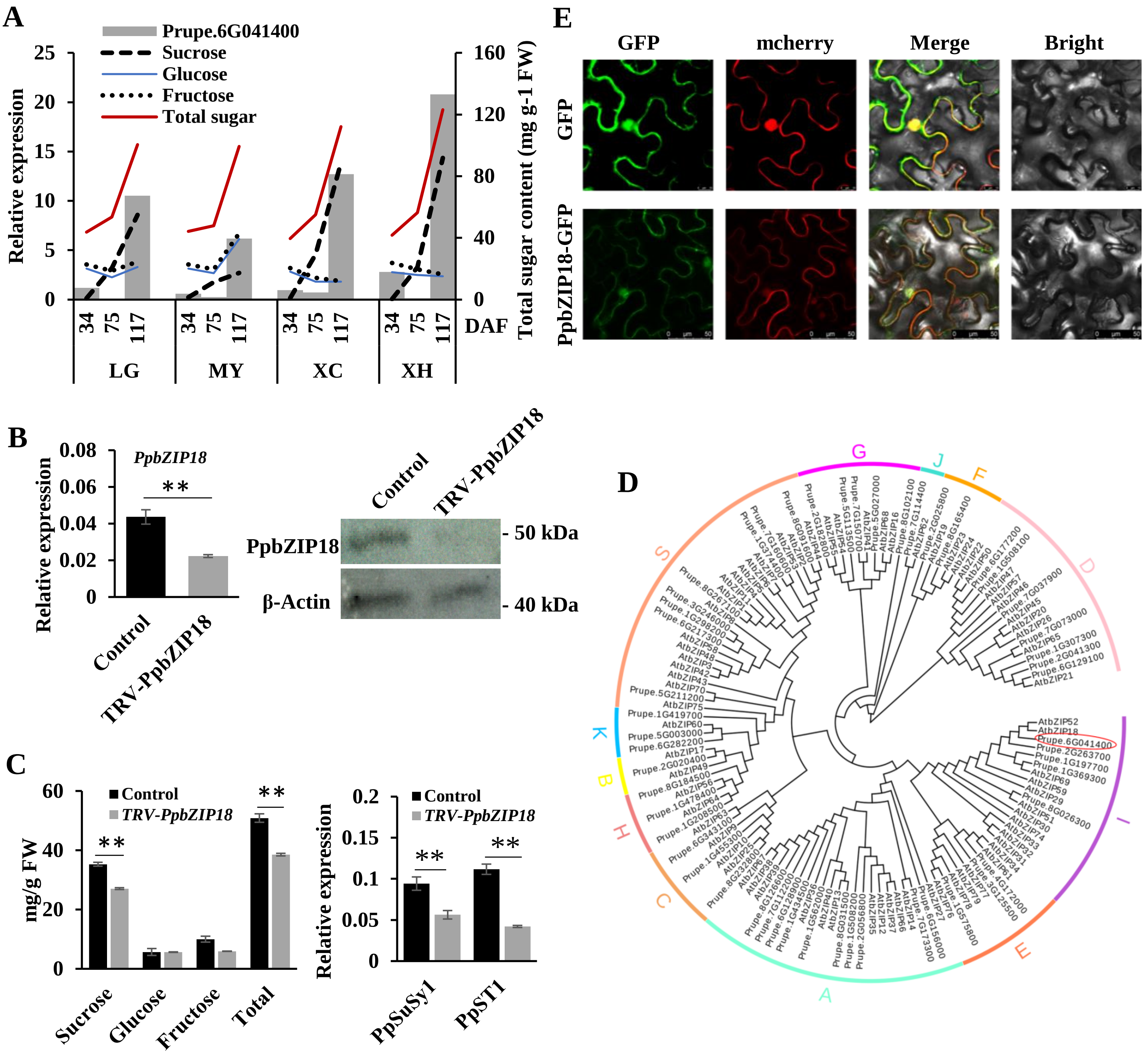

Figure S1 Functional analysis of the role of the *PpbZIP18* gene in sugar accumulation in peach. A. The expression profiles of *PpbZIP18* in fruits of different peach cultivars throughout the development. LG, Ligelante; MY, Meiguowanyou; XC, Xiacui; and XH, Xiahui. DAF represents days after flowering. B. The transcript and protein levels of *PpbZIP18* in *PpbZIP18* transiently silenced fruits of peach C. The content of sugar components and the expression of *PpSuSy1* and *PpST1* in *PpbZIP18* transiently silenced fruits of peach. D. Phylogenetic analysis of bZIP TFs from peach and *Arabidopsis*. The peach PpbZIP18 is highlighted in a red circle. E. Subcellular localization assay of PpbZIP18-GFP in *N. benthamiana*. Error bars represent the standard error (n = 3). Asterisks denote significant differences based on Student's *t*-test. $*P < 0.05$, $**P < 0.01$.

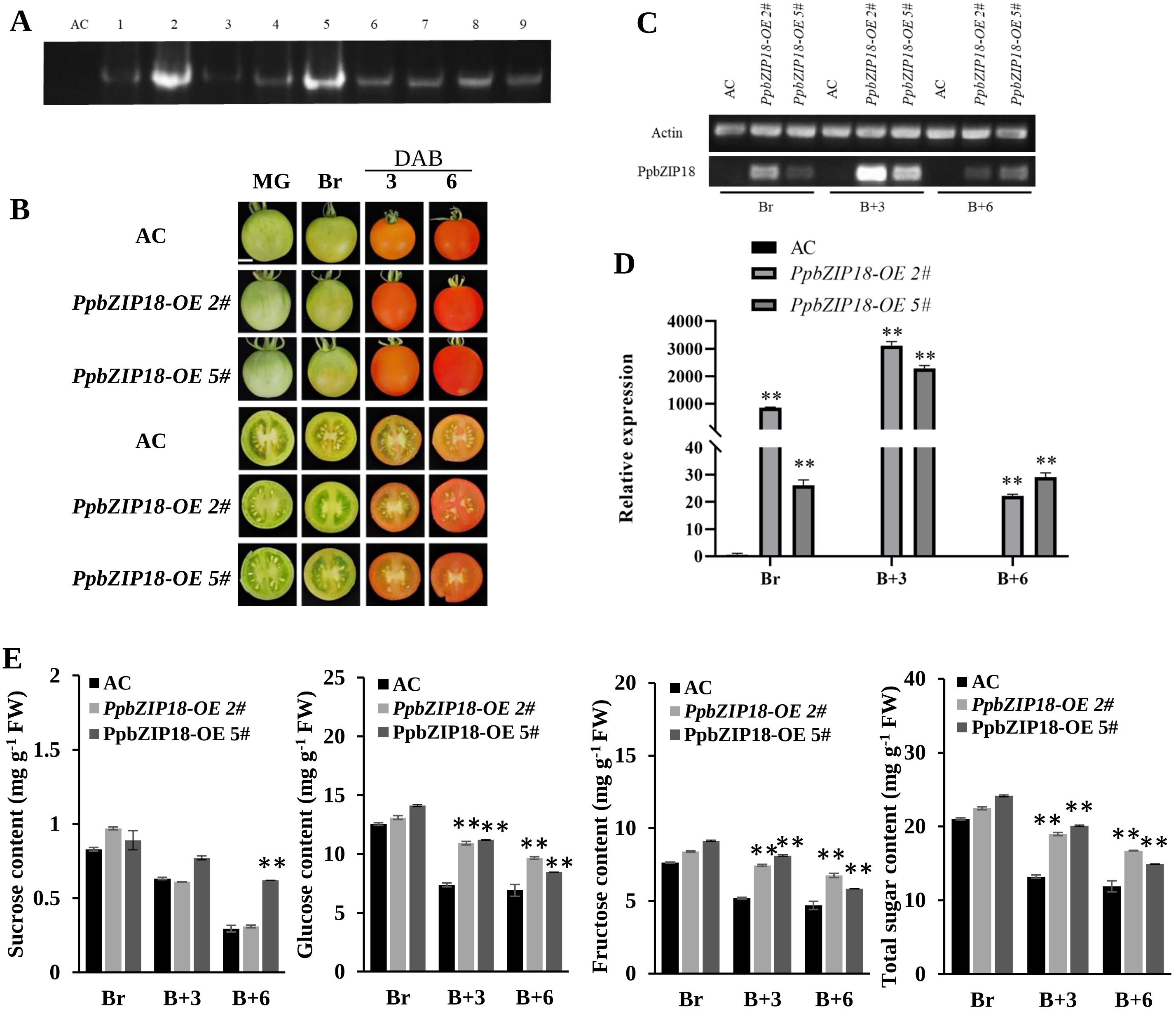

Figure S2 Transgenic tomatoes overexpressing *PpbZIP18*. A. PCR analysis showing the presence of the *PpbZIP18* gene in wild type (AC) and transgenic lines. B. Fruits of tomato 'Alisa Craig (AC)' and transgenic lines at late stages of development. MG, mature green; Br, breaker; and DAB, days after breaker. Bar, 1 cm. C. Analysis of semi-quantitative RT-PCR showing the *PpbZIP18* expression in tomato fruits overexpressing *PpbZIP18* at different developmental stages. D. Quantitative RT-PCR showing the *PpbZIP18* expression in tomato fruits overexpressing *PpbZIP18* at different developmental stages. E. The content of sugar components in WT and transgenic tomato fruits at different developmental stages. Error bars represent the standard error (n = 3). Asterisks denote significant differences based on Student's *t*-test. $*P < 0.05$, $**P < 0.01$.

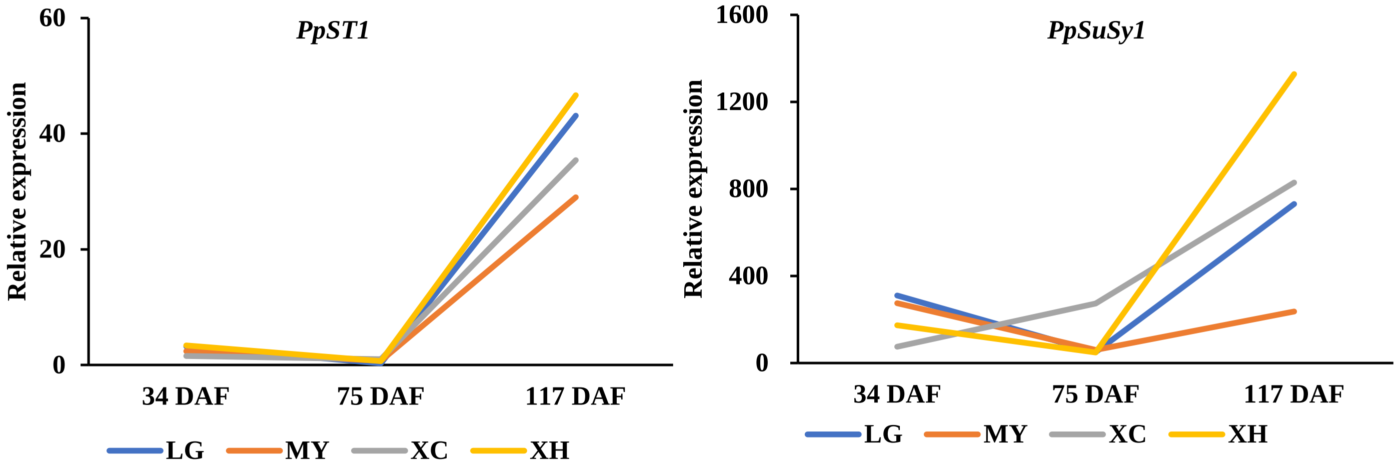

Figure S3 The expression of *PpSuSy1* and *PpST1* in fruits of different peach varieties throughout the development. The expression level of *PpbZIP18* was estimated using our previously reported transcriptome data of cultivated fruits throughout the development (Zheng *et al*., 2021).

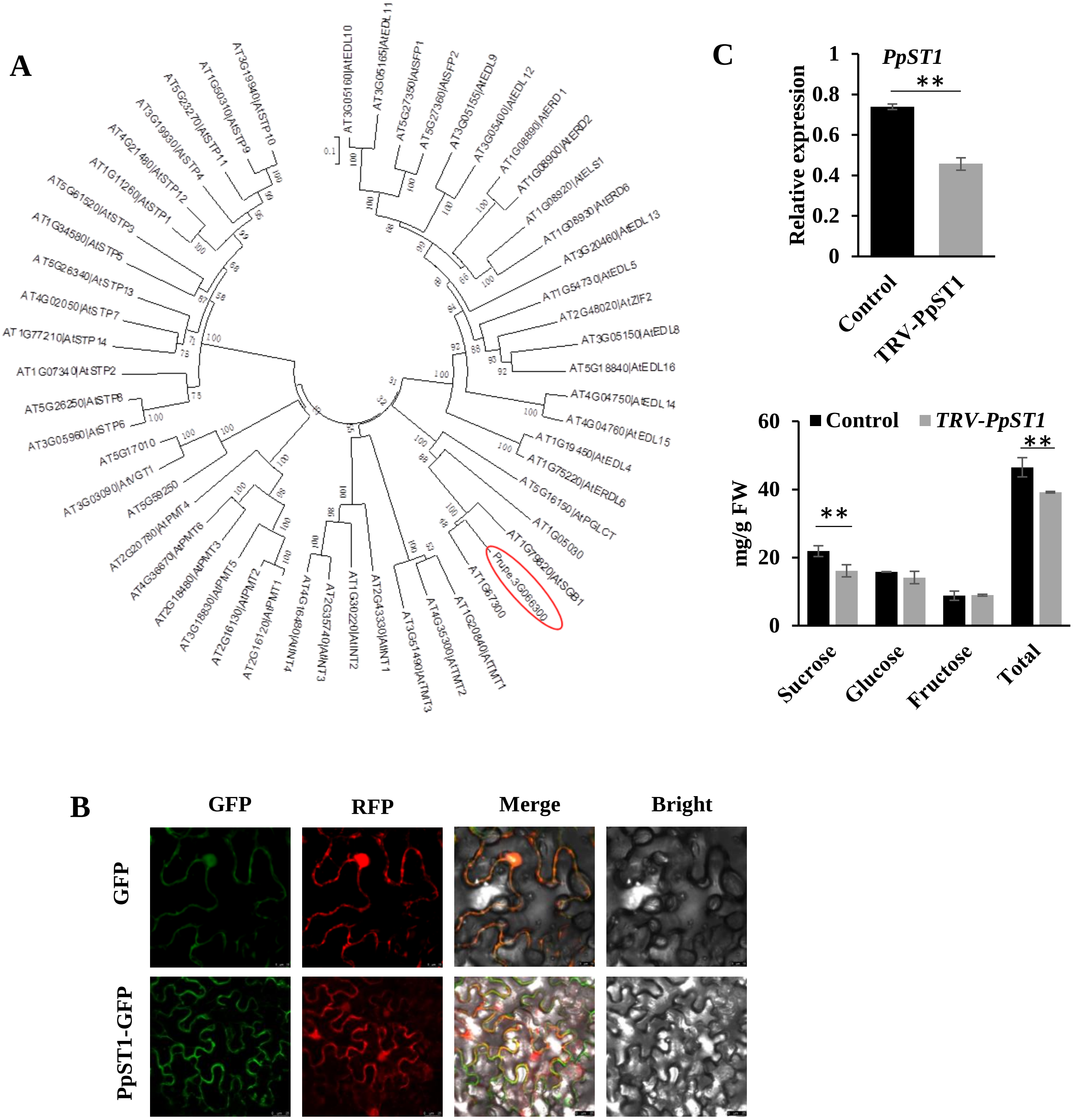

Figure S4 Functional analysis of the role of *PpST1* in sugar transport. A. Phylogenetic analysis of PpST1 and *Arabidopsis* sugar porter (SP) family members. PpSTI is highlighted in a red circle. B. Subcellular localization assay of PpSTI in *N. benthamiana*. C. The expression of *PpST1* and the content of sugar components in *PpST1* transiently silenced fruits of peach. Error bars represent the standard error (n = 3). Asterisks denote significant differences based on Student's *t*-test. **P* < 0.05, ***P* < 0.01.

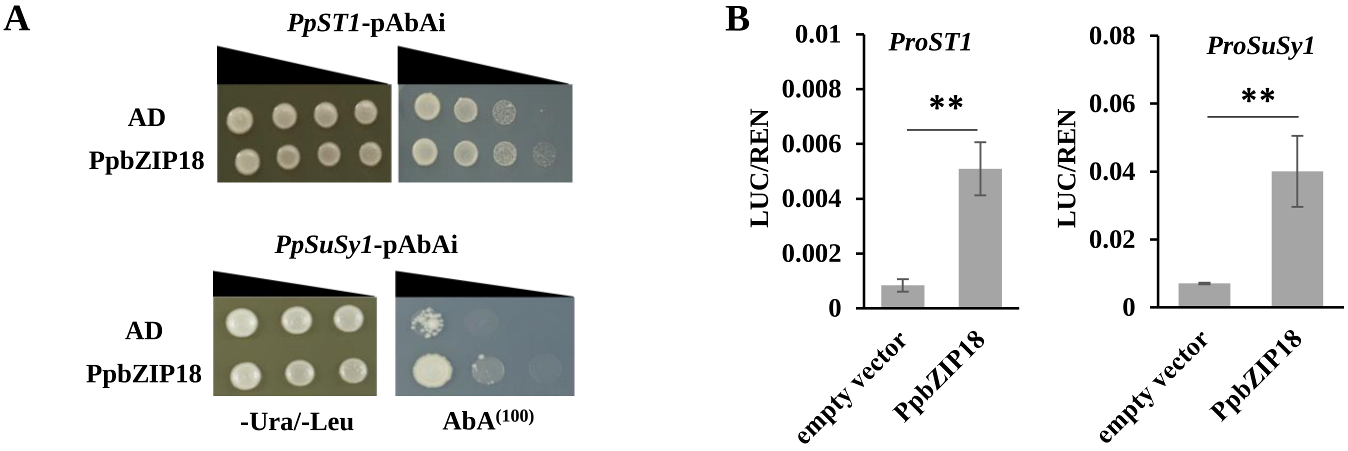

Figure S5 Assay of the binding capacity of PpbZIP18 to the *PpST1* and *PpSuSy1* promoters to activate their expression. A. Yeast one-hybrid assay showing the binding affinity of PpbZIP18 to the promoters of *PpST1* and *PpSuSy1*. B. LUC/REN assay indicating the activation effect of PpbZIP18 on the promoters of *PpST1* and *PpSuSy1*. Error bars represent the standard error (n = 3). Asterisks denote significant differences based on Student's *t*-test. *$P < 0.05$, **$P < 0.01$.

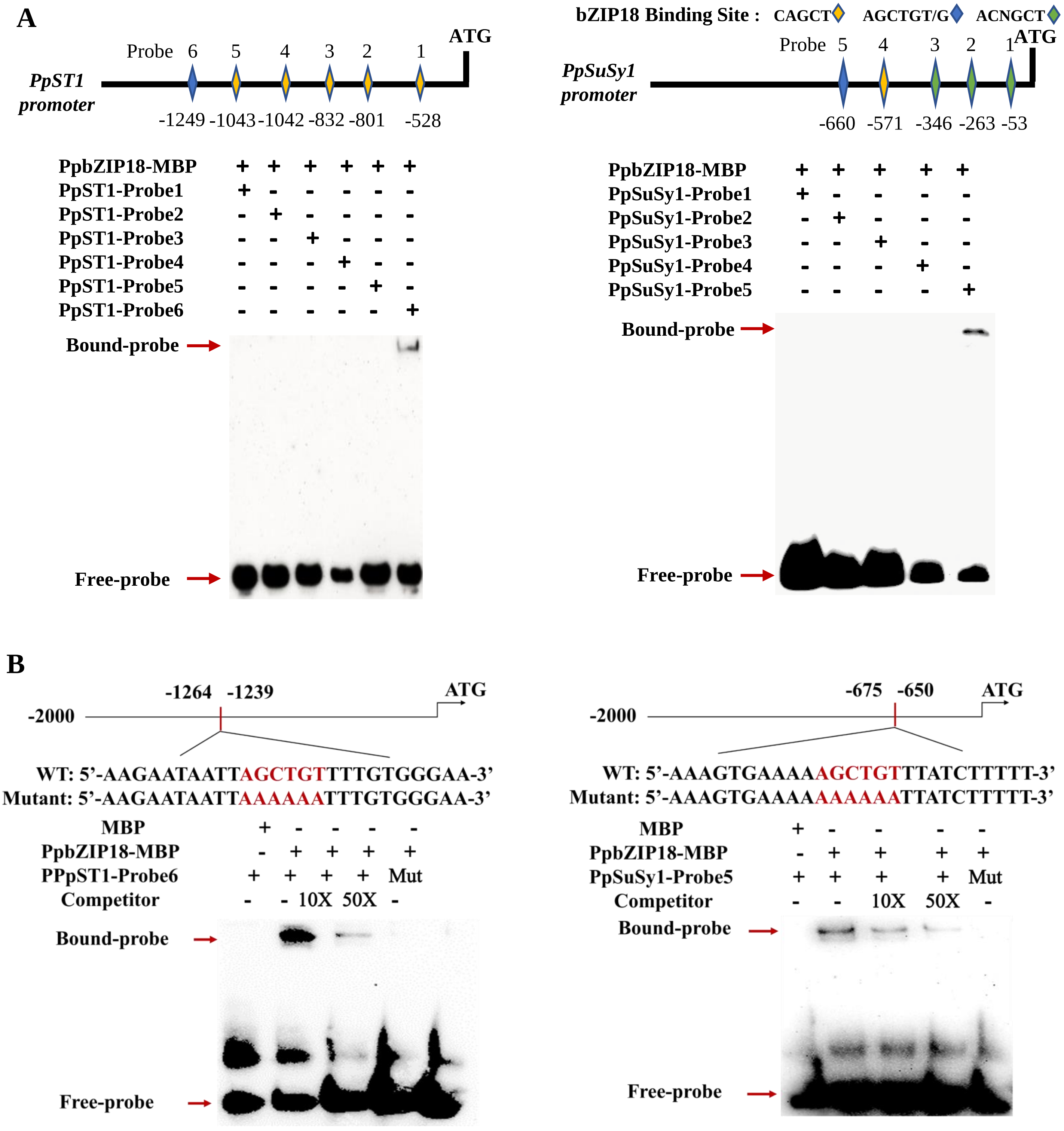

Figure S6 Assay of the binding ability PpbZIP18 to the AGCTGT/G motif in the promoters of *PpST1* and *PpSuSy1*. A. EMSA assay showing the binding motifs of PpbZIP18 in the promoters of *PpST1* and *PpSuSy1*. B. Validation of the binding affinity of PpbZIP18 to the AGCTGT/G motif in the promoters of *PpST1* and *PpSuSy1* using EMSA assay.

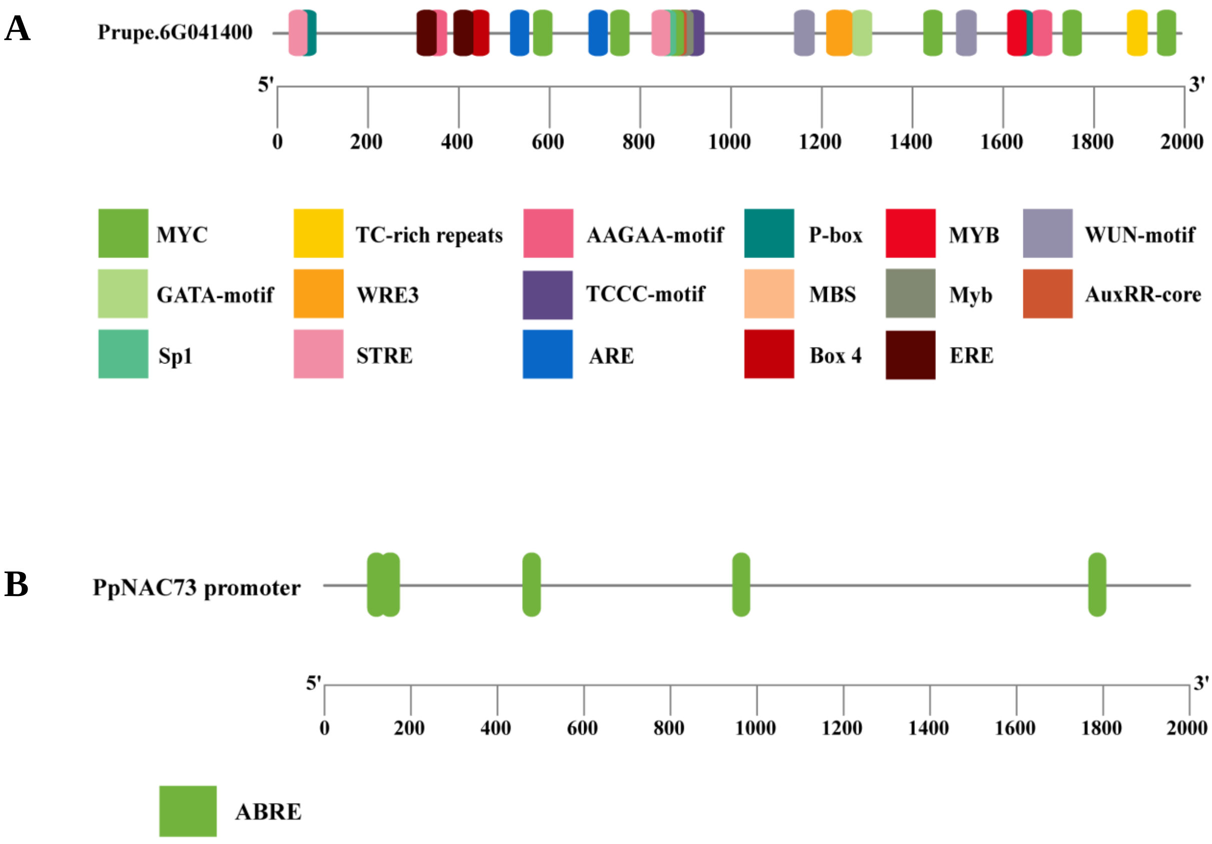

Figure S7 Analysis of the gene promoter using PlantCARE online program. A. The predicted motifs in the *PpbZIP18* promoter. B. The predicted motifs in the *PpNAC73* promoter.

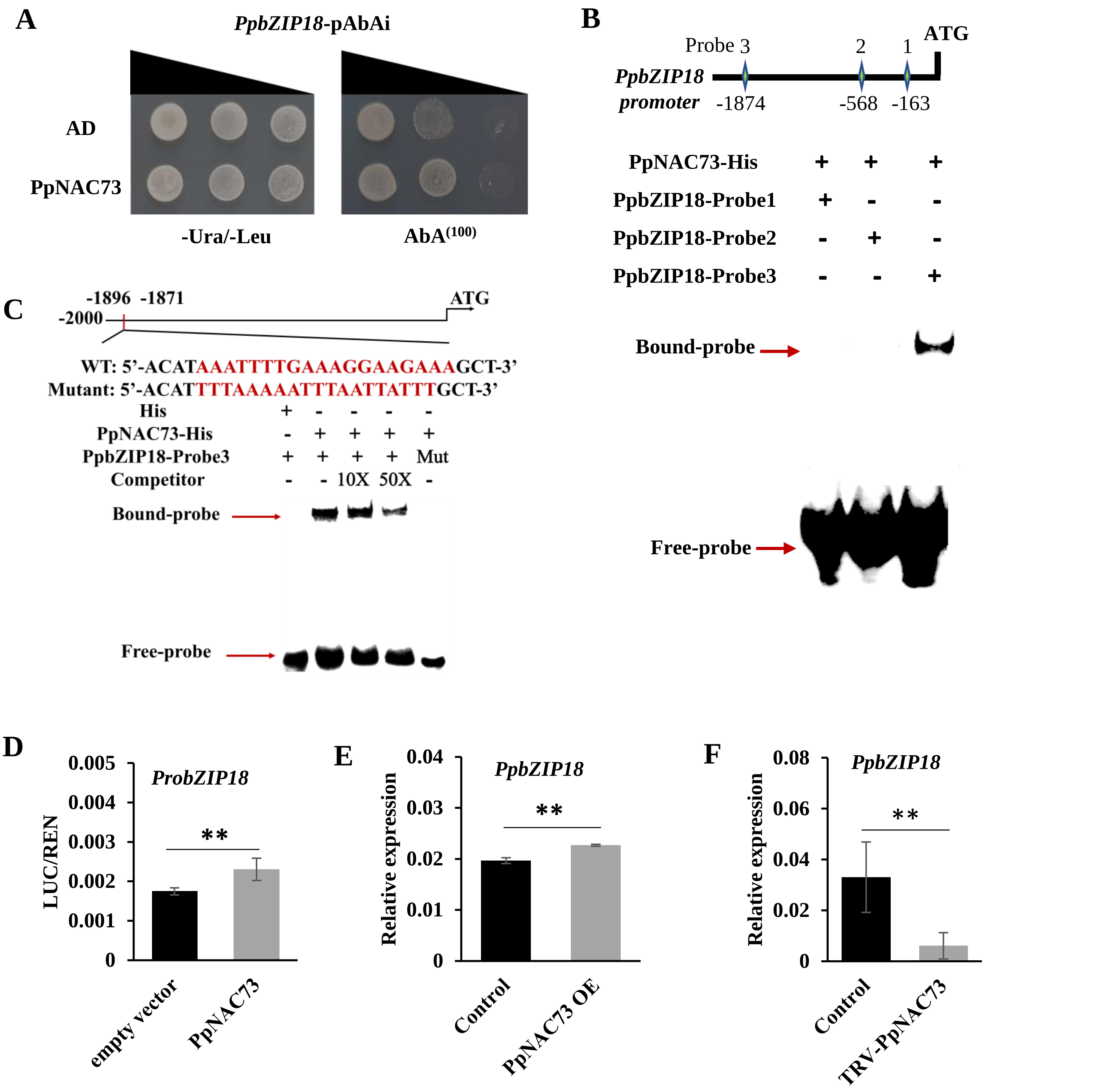

Figure S8 Assay of the binding ability of PpNAC73 to the promoter of *PpbZIP18* to activate its expression. A. Yeast one-hybrid assay showing the binding affinity of PpNAC73 to the *PpbZIP18* promoter. B. EMSA assay showing the binding sites of PpNAC73 in the *PpbZIP18* promoter. C. Validation of the binding affinity of PpNAC73 to the SNBE motif in the *PpbZIP18* promoter using EMSA assay. D. LUC/REN assay indicating the activation effect of PpNAC73 on the *PpbZIP18* promoter. E. The expression of *PpbZIP18* in peach fruits transiently overexpressing *PpNAC73*. F. The expression of *PpbZIP18* in *PpNAC73* transiently silenced fruits of peach. Error bars represent the standard error (n = 3). Asterisks denote significant differences based on Student's *t*-test. *$P < 0.05$, **$P < 0.01$.

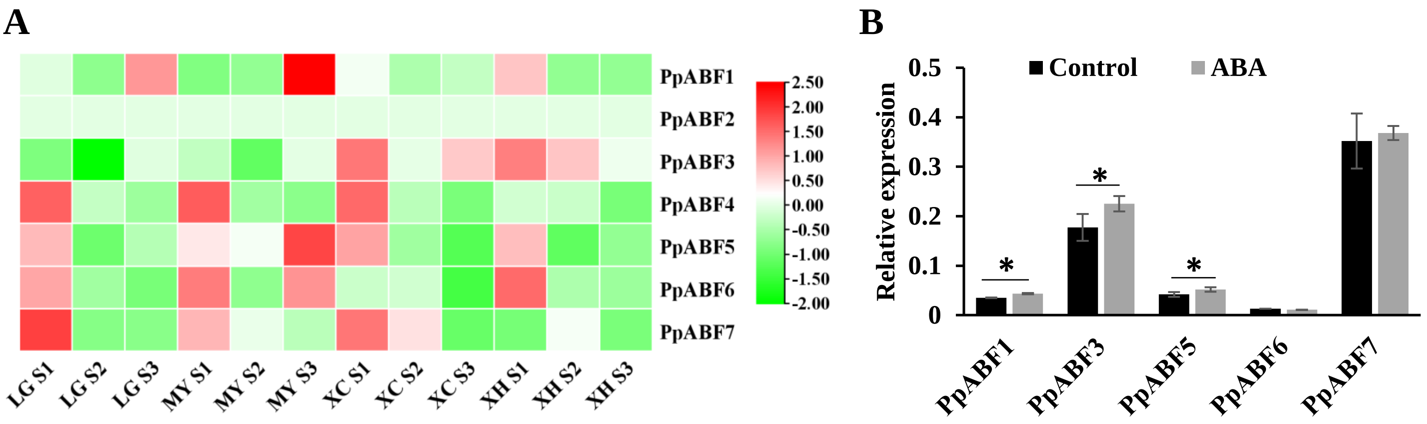

Figure S9 Estimation of the expression profiles of AREB/ABF members in peach fruits. A. The expression levels of *PpABFs* in fruits of different peach varieties throughout the development. S1, 34 days after flowering; S2, 75 days after flowering; and S3, 117 days after flowering. The expression level of genes was estimated using our previously reported transcriptome data of cultivated fruits (Zheng *et al*., 2021). B. Expression levels of *PpABFs* genes in peach fruits at 3 days after ABA treatment. Error bars represent the standard error (n = 3). Asterisks denote significant differences based on Student's *t*-test. *$P < 0.05$, **$P<0.01$. Note: *PpABF2* and *PpABF4* were not expressed in the fruits.

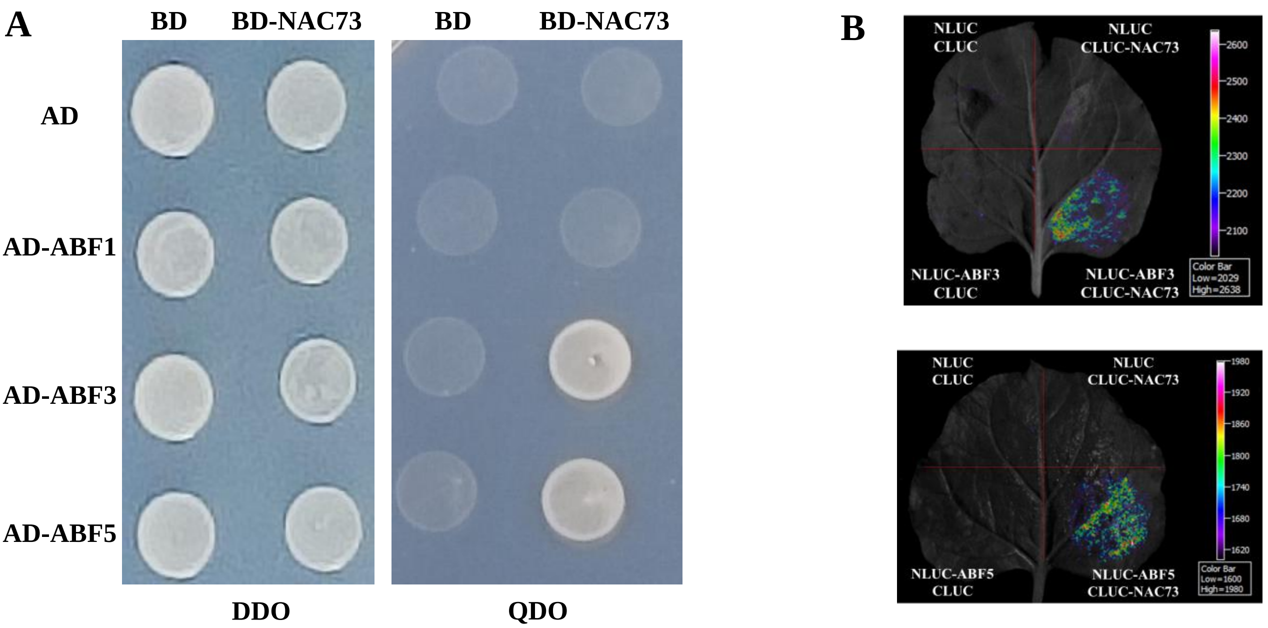

Figure S10 Assay of the interaction between PpABFs and PpNAC73. A. Validation of the interaction between PpABFs and PpNAC73 using Y2H. B. Validation of the interaction between PpABF3/5 and PpNAC73 using the split-luciferase complementation assay in *N. benthamiana*.

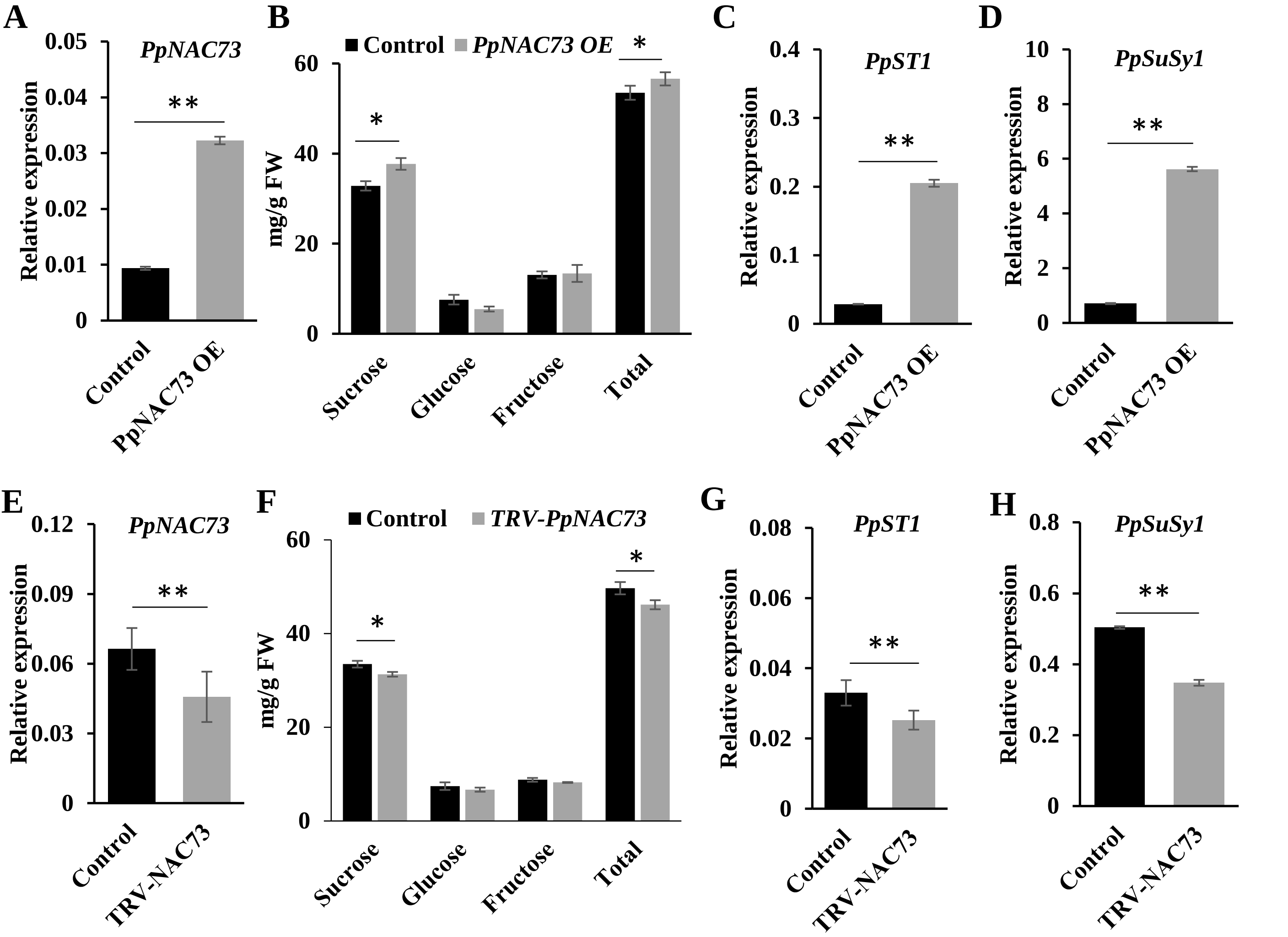

Figure S11 Functional analysis of the role of *PpNAC73* in sugar accumulation using transient transformation assay in peach fruit. A. The expression of *PpNAC73* in peach fruits overexpressing *PpNAC73*. B. The content of sugar components in peach fruits overexpressing *PpNAC73*. C. The expression of *PpST1* in peach fruits overexpressing *PpNAC73*. D. The expression of *PpSuSy1* in peach fruits overexpressing *PpNAC73*. E. The expression of *PpNAC73* in *PpNAC73* silenced peach fruits. F. The content of sugar components in *PpNAC73* silenced peach fruits. G. The expression of *PpST1* in *PpNAC73* silenced peach fruits. H. The expression of *PpSuSy1* in *PpNAC73* silenced peach fruits. Error bars represent the standard error (n = 3). Asterisks denote significant differences based on Student's *t*-test. *$P<0.05$, **$P<0.01$.

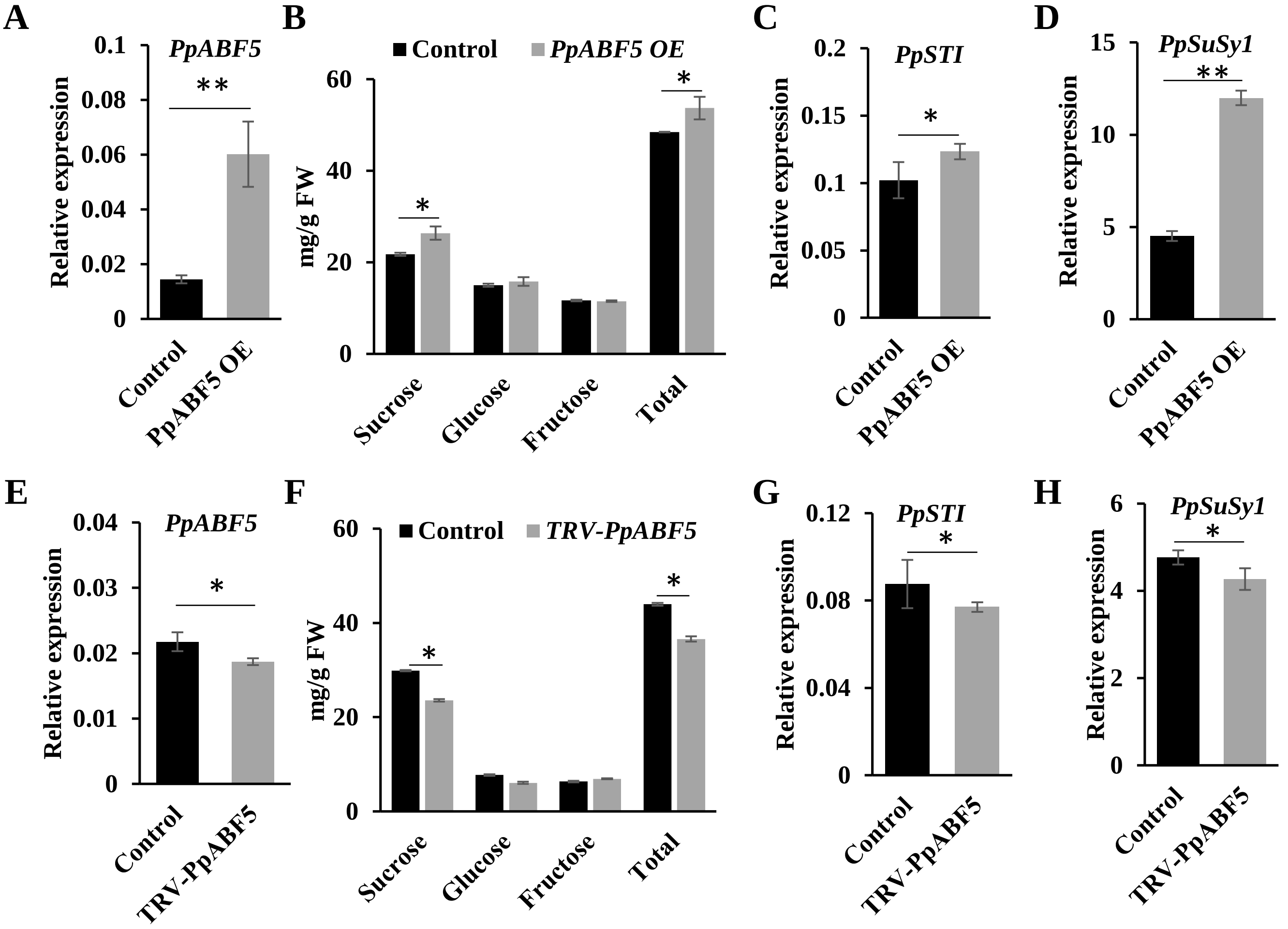

Figure S12 Functional analysis of the role of *PpABF5* in sugar accumulation using transient transformation assay in peach fruit. A. The expression of *PpABF5* in peach fruits overexpressing *PpABF5*. B. The content of sugar components in peach fruits overexpressing *PpABF5*. C. The expression of *PpST1* in peach fruits overexpressing *PpABF5*. D. The expression of *PpSuSy1* in peach fruits overexpressing *PpABF5*. E. The expression of *PpABF5* in *PpABF5* silenced peach fruits. F. The content of sugar components in *PpABF5* silenced peach fruits. G. The expression of *PpST1* in *PpABF5* silenced peach fruits. H. The expression of *PpSuSy1* in *PpABF5* silenced peach fruits. Error bars represent the standard error (n = 3). Asterisks denote significant differences based on Student's *t*-test. *P <0.05, **P < 0.01.

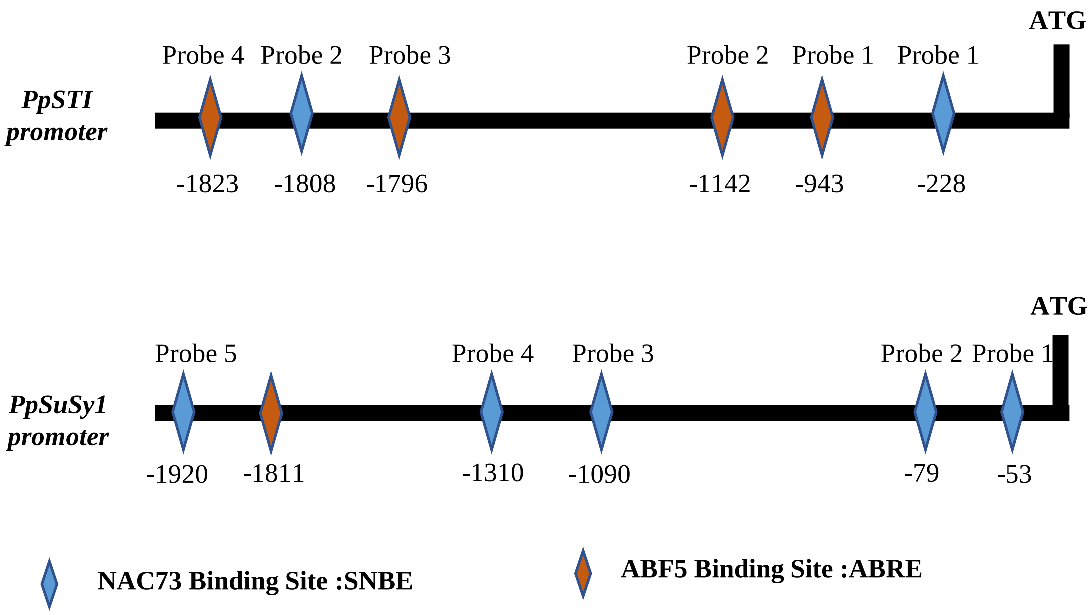

Figure S13 Putative binding sites of PpNAC73 and PpABF5 in the promoters of *PpST1* and *PpSuSy1*.

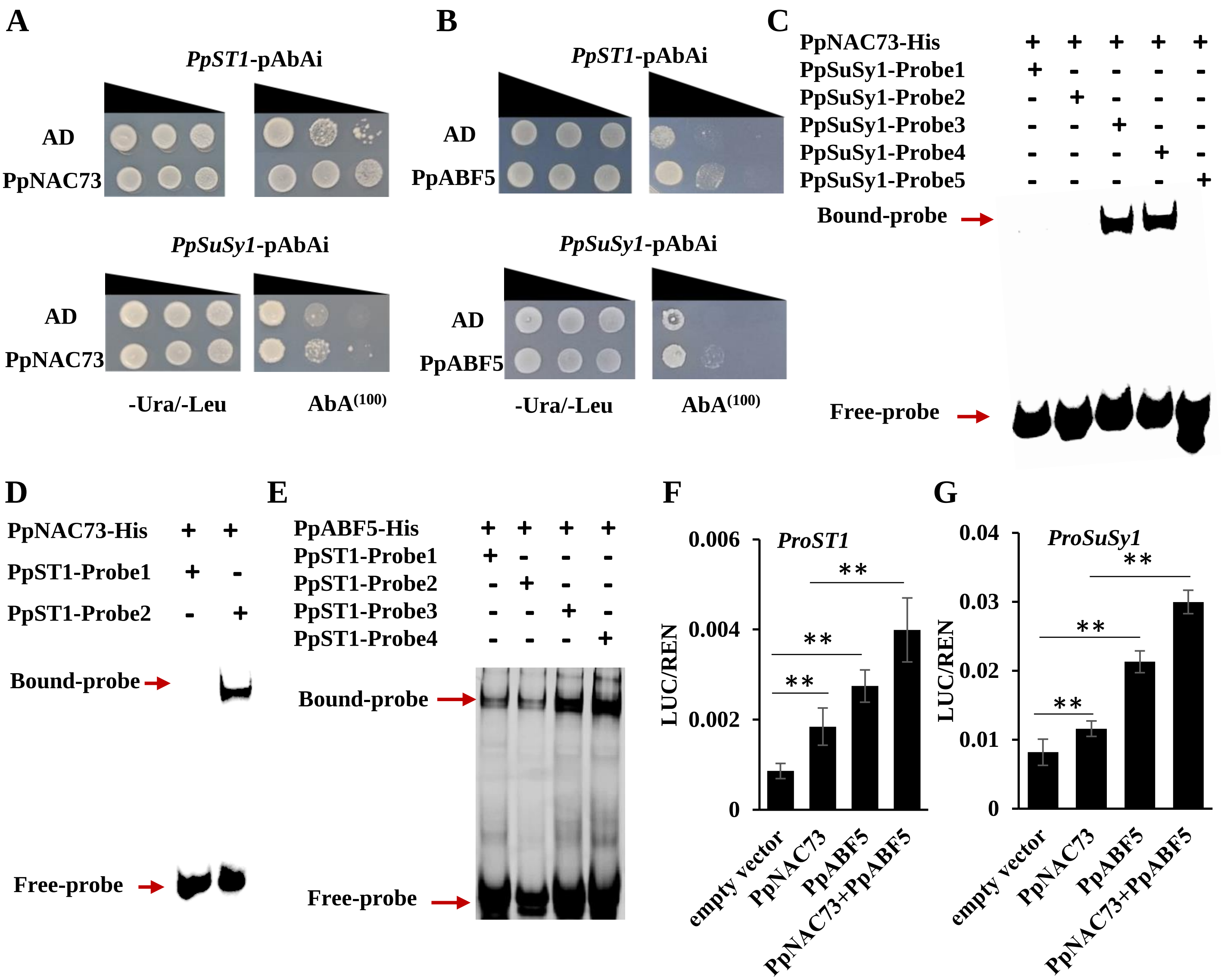

Figure S14 Assay of the binding ability of PpNAC73 and PpABF5 to the promoters of *PpST1* and *PpSuSy1* to activate their expression. A. Yeast one-hybrid assay showing the binding affinity of PpNAC73 to the promoters of *PpST1* and *PpSuSy1*. B. Yeast one-hybrid assay showing the binding affinity of PpABF5 to the promoters of *PpST1* and *PpSuSy1*. C. EMSA assay showing the binding sites of PpNAC73 in the *PpSuSy1* promoter. D. EMSA assay showing the binding sites of PpNAC73 in the *PpST1* promoter E. EMSA assay showing the binding sites of PpABF5 in the *PpST1* promoter. F. LUC/REN assay showing the activation effect of PpNAC73 and PpABF5 on the *PpST1* promoter. G. LUC/REN assay indicating the activation effect of PpNAC73 and PpABF5 on the *PpSuSy1* promoter. Error bars represent the standard error (n = 3). Asterisks denote significant differences based on Student's *t*-test. *$P < 0.05$, **$P < 0.01$.

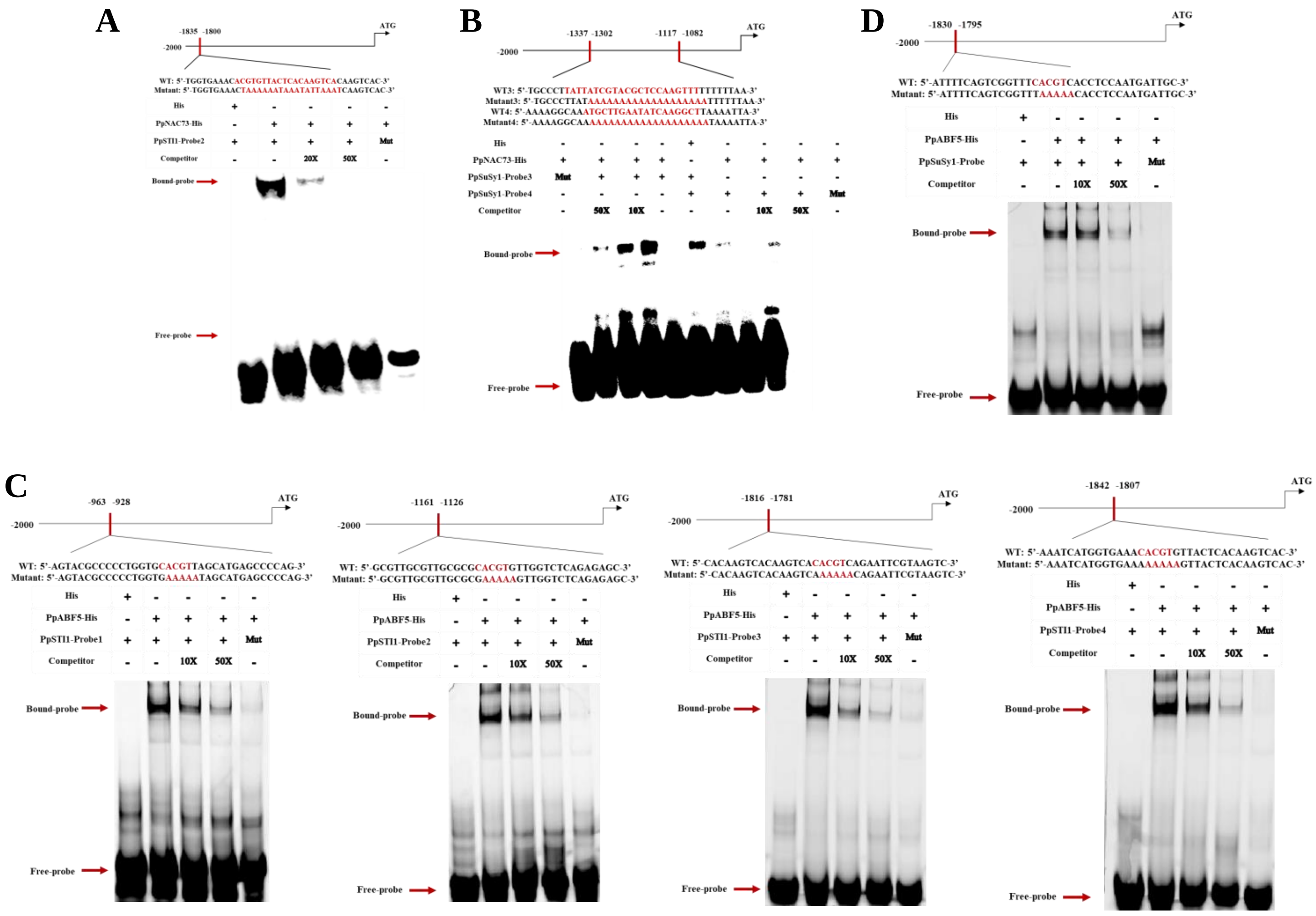

Figure S15 EMSA assay indicating the binding capacity of PpNAC73 and PpABF5 to the motif in the *PpST1* and *PpSuSy1* promoters. A. The binding capacity of PpNAC73 to the SNBE motif in the *PpST1* promoter. B. The binding capacity of PpNAC73 to the SNBE motif in the *PpSuSy1* promoter. C. The binding capacity of PpABF5 to the ABRE motif in the *PpST1* promoter. D. The binding capacity of PpABF5 to the ABRE motif in the *PpSuSy1* promoter.
