# Supplementary material for: A subgroup I bZIP transcription factor PpbZIP18 plays an important role in sucrose accumulation in peach
Source: Mol Hortic. 2025 Jul 3;5:36. doi: 10.1186/s43897-025-00156-0 (PMC12224830; doi:10.1186/s43897-025-00156-0)
Supplement: Supplementary file 3 — Supplementary Material 3. [file 43897_2025_156_MOESM3_ESM.docx]

**A subgroup I bZIP transcription factor PpbZIP18 plays an important role in sucrose accumulation in peach**

Xian Zhang^1,2,3^, Wen Xiao^1,4^, Yudi Liu^1,3^, Yunpeng Cao^1^, Ruo-Xi Zhang^1*^, Yuepeng Han^1,2*^

^1^State Key Laboratory of Plant Diversity and Specialty Crops, Wuhan Botanical Garden, Chinese Academy of Sciences, Wuhan 430074, China

^2^ Hubei Hongshan Laboratory, Wuhan 430070, China

^3^ University of Chinese Academy of Sciences, Beijing 100049, China

^4^ State Key Laboratory of Hybrid Rice, Department of Plant Sciences, College of Life Sciences, Wuhan University, Wuhan 430072, China

*Correspondence author

**Materials and methods**

**Plant materials**

Peach fruits used in this study were collected from the peach filed of Wuhan Botanical Garden of the Chinese Academy of Sciences. *Nicotiana benthamiana* seedlings were grown in a plant growth chamber at 24°C with a light/dark cycle of 16 h/8 h and a relative humidity of 70% ± 5%.

**ABA treatment of peach fruits**

Peach fruits of 'Jinxiu' at the second stage of enlargement were selected for ABA experiment. The selected fruits were of similar size and had no any visible damage. For the ABA treatment, fruits were immersed in an ABA solution with a concentration of 100 mg/L, ensuring that the fruits were completely submerged in the solution for 10 minutes. Fruits immersed in deionized water were used as the control. After the treatment, the fruits were gently patted dry with a paper towel, and then placed in a lighted incubator. Samples were taken at 0 d, 1 d, 3 d, and 6 d after treatment, with at least three fruits per sampling point. The peel was removed, and the pulp was extracted and stored in a -80°C freezer for subsequent experiments.

**Measurement of soluble sugars**

Approximately 1g of fruit samples was ground into a powder using liquid nitrogen. The powder was mixed with 5 mL of deionized water (ddH_2_O). The mixture was sonicated for 15 minutes at 4°C, and then centrifuged at 5000 rpm for 15 minutes at 4°C. The supernatant was transferred to a new 1.5 mL centrifuge tube and filtered three times with a 0.22 μm filter membrane. The filtered supernatant was subjected to soluble sugar quantification using high-performance liquid chromatography (HPLC). The analysis was performed using an Agilent 1260 HPLC system (Agilent, Karlsruhe, Germany) equipped with a refractive index detector and a Carbo Sep CHO-620 Ca sugar analysis column (Catalog No.: LAAO-C9753, Transgenomic) along with a guard column (Catalog No.: LBAO-C2353, Transgenomic). Ultrapure water was used as the mobile phase, with a flow rate of 0.5 mL/min and a column temperature of 90°C.

The standard curve for each sugar component was obtained by determining the peak areas corresponding to different concentrations. The obtained peak areas from HPLC analysis of each sample, along with the standard curve, were used to calculate the concentrations of soluble sugars. The concentrations of soluble sugars are expressed in mg/g FW (milligrams per gram fresh weight).

**Bioinformatics analysis**

Gene sequences from peach and *Arabidopsis thaliana* were retrieved from GDR (https://www.rosaceae.org) and TAIR (https://www.arabidopsis.org/), respectively. Neighbor-joining phylogenetic trees were constructed using MEGA 6. To ensure the reliability of the tree topologies, 1,000 bootstrap replicates were used.

**Assay of subcellular localization**

The entire coding sequences (CDS) without stop codons of *PpbZIP18*, *PpST1* or *PpNAC73* were individually inserted into psuper1300-GFP vector and then transformed into Agrobacterium GV3101. Agrobacterium suspension containing the gene of interest was mixed with that containing plasma membrane markers or vacuolar membrane markers in a ratio of 1:1 (V/V). The mixture was injected into tobacco leaves of 4-week-old plants. Three days after transformation, GFP fluorescence was detected using a laser confocal 3D scanner (Leica/Germany, TCS SP8) with excitation/emission wavelengths of 488/610 nm. The primers are listed in Table S1.

**Transient transformation assay in peach fruit**

For transient overexpression assay, Agrobacterium cultures containing the gene of interest were adjusted to an OD600 of 0.6 to 0.8 with infiltration buffer (10 mM Mgcl_2_, 20 mM MES, 150 μM AS，PH=5.6). The adjusted solution was injected into one side of the fruit using a 1 mL syringe, while the opposite side of the same fruit was injected with empty vector as the control. For each treatment, at least three biological replicates were conducted. After injection, the fruits were placed in a growth chamber under light conditions, and were sampled 3 days later.

For transient silencing assay, a 300 bp fragment of *PpbZIP18, PpST1* or *PpNAC73* was individually inserted into pTRV2. The construct was transferred into strain GV3101. Infiltration buffer containing pTRV1 was mixed with that containing pTRV2-construct in a 1:1 ratio. The mixture was injected into one side of the fruit, while the opposite side of the same fruit was injected with pTRV1 and pTRV2 as the control. Peach fruits used for transient transformation assay were collected from ‘Jinxiu’ at the stage of the second exponential growth. The primers are listed in Table S1.

**Western blot assay**

Western blot was used to detect the level of bZIP18 protein in transiently transformed fruits. Briefly, total proteins were extracted from transiently transformed fruits using Plant Fruit Protein Extraction Kit (Wanleibio, Shenzhen) according to the manufacturer’s instructions. After separation by SDS-PAGE electrophoresis, the proteins were transferred onto a PVDF membrane and subsequently blocked with 5% skimmed milk powder to prevent non-specific binding. The membrane was then incubated overnight at 4°C with primary antibodies (β-Actin diluted 1:5000, PpbZIP18 diluted 1:100), followed by incubation with secondary antibody (diluted 1:5000) for 1 hour. Finally, the membrane was placed in ECL luminescent solution and visualized using a chemiluminescent imaging system GelView9000 Lite (Guangzhou Biolight Biotechnology Co., Ltd.) to detect the target proteins.

**Tomato stable transformation**

Tomato seeds were sterilized, sown on 1/2 MS medium, and then placed in a growth chamber under light conditions for germination. When the seeds germinated and the cotyledons unfolded, the cotyledons were excised and soaked in MS liquid medium for 1 hour. Later, the excised cotyledons were placed on a pre-culture medium for 1 day in a dark condition. The vector containing the gene of interest was transformed into the GV3101 Agrobacterium tumefaciens strain, and the resulted Agrobacterium culture was used to infect the pre-cultured cotyledons for 15 minutes. The infected cotyledons were then placed on MS medium. After 2 days of culture, the cotyledons were transferred to antibiotic-containing medium and cultured at 25°C under a photoperiod of 16 hours of light/8 hours of darkness. Once callus formation occurs, transformants were transferred to subculture medium to induce adventitious shoots. Finally, the transformants were placed on rooting medium for root formation. Positive transformants were identified and selected for subsequent experiments.

**Yeast one-hybrid assay (Y1H)**

Yeast one-hybrid assays were performed to test the interaction between transcription factors and the promoters of genes of interest using the Yeastmaker^TM^ Yeast Transformation System 2 kit (TaKaRa, Japan). The promoter sequences of *PpSuSy1*, *PpST1* or *PpbZIP18* were individually amplified and inserted into the pAbAi vector. The full-length coding sequences of *PpbZIP18* or *PpNAC73* were cloned into the pGADT7 vector. The PpTFs-pGADT7 plasmid was transformed into the Y1HGold strain carrying the promoter sequences of genes of interest, and the empty vector pGADT7 was used as negative control. The primers are listed in Table S1.

**Dual-luciferase reporter assay**

The full-length coding sequences of transcription factors were amplified and inserted into the pSAK277 vector. The promoters of genes of interest were inserted into the pGreen II 0800-LUC vector. Primers for vector construction are listed in Table S1. The constructed pSAK277 and pGreenII 0800-LUC vectors containing the genes of interest were transformed into Agrobacterium tumefaciens strains GV3101 and GV3101+psoup+p19, respectively, and the cultures were adjusted to an OD600 of 1.5 with infiltration buffer (10 mM MgCl_2_ and 150 μM AS). *A. tumefaciens* containing TF was mixed with that containing the promoter of gene of interest in a 4:1 ratio. The mixture was infiltrated into tobacco leaves by a syringe. Three days after infiltration, discs from the tobacco leaves were collected, and the enzyme activities of firefly luciferase (LUC) and Renilla luciferase (REN) were measured on a GloMax 96 Microplate Luminometer (TECAN, M200 PRO). At least 4 biological replicates were performed for each experiment.

**Electrophoretic mobility shift assay (EMSA)**

EMSA was performed using the Chemiluminescent EMSA Kit (Beyotime, China) according to the manufactures’ instructions. Primers for EMSA experiments are listed in Table S2. Briefly, biotin-labeled probes were developed from the promoters of *PpSuSy1*, *PpST1* or *PpbZIP18*. These probes were subsequently incubated with protein in a binding solution for 30 minutes at room temperature. Later, all reactants were electrophoresed on a nondenaturing 6.7% polyacrylamide gel at 120 V and 4°C for 1 hour. After electrophoresis, the gels were transferred onto Biodyne B nylon membranes and cross-linked using UV light. Subsequently, the nylon membranes were eluted with Streptavidin-HRP and BeyoECL Moon reagent, and ultimately visualized using a multifunctional imaging system (FluorChem R, USA).

**Heterologous expression of *PpST1* in yeast**

For complementation assay in yeast cells, the coding sequence of *PpST1* was inserted into the PDR196 vector. Primers for vector construction are listed in Table S1. The construct was transformed into the hexose transport- and SUC2-deficient yeast strain CSY4000. The CSY4000 strain was provided by Professor Chunlong Li of Huazhong Agricultural University. The empty pDR196 vector was used as a negative control. The transformants were cultured on SD/-ura media supplemented with maltose/sucrose/glucose/fructose as the sole carbon source. Yeast cells were grown on medium for 3 to 4 d at 30°C.

**Yeast two-hybrid assay (Y2H)**

The constructed bait (pGBKT7) and prey (pGADT7) vectors were simultaneously transferred into the Y2H yeast strain and then screened on selective medium containing X-α-gal. The interaction between the bait and prey proteins was indicated by appearance of blue color in the strain that was incubated on QDO+X-α-gal medium.

**Split-luciferase complementation assay**

The split-luciferase complementation assay (NC-LUC) was conducted following a previously reported method (Zhang et al., 2024b). Briefly, the firefly LUC protein was divided into its N-terminal part (nLUC) and C-terminal part (cLUC). The entire coding sequence (CDS) of *PpABFs* was cloned into the pCAMBIA-nLUC vector, whereas the full-length CDS of *PpNAC73* was inserted into the pCAMBIA-cLUC vector. The primers used for vector construction are detailed in Table S1. Agrobacterium strains harboring the indicated constructs were mixed in a 1:1 ratio, and the mixture was used to infiltrate *N. benthamiana* leaves. After infiltration, the plants were placed in a growth room for three days. Subsequently, the infiltrated leaves were sprayed with Luciferin (1 mM) and kept in the dark for 10 minutes. Luminescence detection was then performed using a low-light cooled CCD imaging apparatus (Andor iXon, Grens, Switzerland).

**Co-immunoprecipitation assay (Co-IP)**

A Co-IP assay was performed according to a previously reported protocol (Wang et al., 2023) with minor modification. Briefly, the coding sequences of *PpABF5* and *PpNAC73* were individually inserted into the PQB vector. Then, PQB-PpABF5 and PQB-PpNAC73 were inserted into pGWB17-MYC and pK7FWG2.0-GFP expression vectors, respectively. The recombinant constructs were transformed into Agrobacterium, which were subsequently used to infiltrate *N. benthamiana* leaves. The infiltrated tobacco leaves were sampled at 3 days after infiltration and then subjected to total protein extraction. Anti-GFP (Green Fluorescent Protein) antibody-bound agarose beads (MBL, cat. no. D153-8) was used to immunoprecipitate the PpNAC73-GFP and GFP proteins. The separation of the resulting protein samples was performed using SDS-PAGE gel electrophoresis and then detected using immunoblotting with anti-GFP (Abmart, cat. no. M20004) and anti-MYC (Abmart, cat. no. M20002) antibodies. Primers are listed in Table S1.

**Statistical analysis**

All assays were performed with three biological replicates. All data in this research are presented as Mean ± S.E. The statistical significance of differences between means was tested using Student's *t*-test (**P* < 0.05, ***P* < 0.01).

**Reference**

Wang J, Li C, Li L, et al. DIW1 encoding a clade I PP2C phosphatase negatively regulates drought tolerance by de-phosphorylating TaSnRK1.1 in wheat. J Integr Plant Biol. 2023;65(8):1918-1936.

Zhang RX, Liu Y, Zhang X, et al. Two adjacent NAC transcription factors regulate fruit maturity date and flavor in peach. New Phytol. 2024;241(2):632-649.
